# Münchausen’s Syndrome in the Form of Factitious Vomiting in a Young Female

**Published:** 2011

**Authors:** Babak Hemmatian Borojeni, Seyyed Mohammad Salar Zaheriany, Nahid Hemmatian Borojeni, Reza Bidaki

**Affiliations:** 1 Undergraduate Clinical Psychology student, Department of Psychology, University of Tehran;; 2 Undergraduate Medical Student, Tehran University of Medical Sciences,; 3 Associate Professor and Chief of Psychiatry service, Rafsanjan University of Medical Sciences.

**Keywords:** Factitious disorders, Münchausen’s syndrome, Recurrent Vomiting, Sick Role

## Abstract

This case report describes a 34-year-old female patient who suffered from chronic vomiting. Different diagnostic and therapeutic procedures were noncontributory. Eventually, psychiatric interviews specified that she has a dependent, self-hurting and attention-seeking personality. She wanted to assume the “sick role”. Malingering and conversion disorder were ruled out and Münchausen’s syndrome was proposed. She received sertraline, emotional ventilation, and cognitive-behavioral therapy and her symptoms resolved.

## Introduction

Every year a considerable amount of time and money is spent to treat patients with factitious disorders who make extensive use of medical services without there being a medical situation to deal with (1). Factitious disorders are physical or psychological symptoms that are intentionally produced in order to assume the sick role. Nevertheless, these are quite different from malingering in the fact that their main intention is to assume the sick role. 

An extreme form of factitious disorder is known as Münchausen’s syndrome in which individuals induce physical illnesses in themselves or others (in which case it is called Münchausen’s syndrome by proxy). This disorder is notoriously difficult to diagnose and it is usually after thorough medical examinations by different physicians that the disorder begins to become overt (2). Finding measures to identify these patients would help sparing a lot of time for medical specialists and a better use of social resources. This case report sets out to make a contribution, as small as it may be, in that respect.

## Case Report

The case was a 34-year-old female worker who presented in May 2010 as a new patient upon referral from her doctor. Her chief complaint was an eight-month history of recurrent vomiting with no apparent association with eating. She had visited different physicians for her problem and different anti-emetics were prescribed but no recovery was seen. In addition to vomiting several times a day, she also complained of a vague abdominal pain and pain in her spine and hands. 

She was grief-stricken due to death of her mother one year before to whom she was much attached. She also lost one of her brothers in a car accident 8 years before.

Due to the worsening of the illness, she was referred to a more supplied center and different procedures including upper GI endoscopy, colonoscopy and barium enema were performed. All these procedures were normal. Her socioeconomic status was considered as low and she worked at a halfway restaurant. Laboratory assays including complete blood count (CBC), serum albumin, blood urea and electrolytes such as serum K^+ ^level were measured and nothing abnormal was found. No organic cause was identified, her vomiting was attributed to a psychogenic problem and she was referred to our psychiatry service.

*Psychiatric evaluation*

In her first psychiatric interview she did not speak clearly and did not establish an appropriate eye contact. Gradually, during the interview, her voice became clearer, but “Double Messages” emerged, her speech being irrelevant to her nonverbal signs. She talked freely about her symptoms, making malingering an unlikely diagnosis.

During three sessions of psychiatric evaluation, she pointed to physical abuse in her childhood. She has had no seriously ill family member in childhood and any friend or relative with alike symptoms. She presented that after her mother’s death, nobody pays attention to her and nobody loves her. Her relationship with her husband was also poor and extramarital relationships were seen as a possibility during the interview. She stated that she was careless about her appearance and weight. She was seen to have a sensitive, irritable, relatively dependent and self-hurting character with a craving for attention.

Findings during the interviews included nervousness and tremor, but no delusion, hallucination, sleep disorder, or obsessive-compulsive disorder (OCD). Signs of depression like dysphoric mood, anhedonia, suicidal thoughts, and loss of appetite and feeling of worthlessness were present.

Eventually, she pointed out that she was manipulating her pharynx to cause vomiting and attract the attention of others. This manipulation continued into a stage that she reported her vomit became bloody. 

The patient had no secondary gain, but primary gain was achieved. She wanted to assume the “sick role”. So malingering, conversion disorder and organic problems were ruled out and an extreme form of factitious disorder known as Münchausen’s syndrome was proposed. 

*Treatment and Follow-up*

Sertraline (50 mg/day) was prescribed for her. She was bedridden in hospital for 9 days and during this period, emotional ventilation was performed. Although she had few visitors, she responded well to treatment, as the frequency and severity of her vomiting declined and her mood improved. Then she was referred for psychotherapy including supportive psychotherapy and cognitive-behavioral therapy (CBT) which were beneficial for her.

## Discussion

Vomiting is a nonspecific symptom. It is a forceful ejection of upper gut contents from the mouth that is usually transient. Vomiting has 2 general etiologies:

1. *Organic***: **

a) Medications such as nicotine, digoxin, opiate analgesics, non-steroidal anti-inflammatory drugs, and erythromycin (3).

b) Gastrointestinal diseases such as mechanical obstruction, antral web, etc (4). 

c) Infections (5) and toxins (6)

d) CNS causes such as increased intracranial pressure

e) Hormonal and metabolic sources such as uremia and diabetes mellitus

2. *Psychiatric*:

The most common psychiatric etiologies for vomiting are somatoform and factitious disorders.

Since vomiting in this case had a chronic course which lasted for about 8 months and according to noncontributory findings of the physical examinations and procedures performed for her, acute etiologies of vomiting such as infection, toxins, and endogenous toxins including uremia and diabetic acidosis were ruled out. The patient did not show any symptoms of scleroderma or jaundice, so liver problems were ruled out as well. The bowel sounds were normal, so mechanical obstruction was not the case and eventually nothing abnormal was observed in her neurological examination. These findings were in favor of chronic causes of vomiting like long-standing medications or psychiatric conditions. The patient did not report weight loss, so it wasn’t necessary to check the serology markers (7).

About the motivations in the factitious disorders it should be noted that assuming the sick role is a way to decrease anxiety and pressures related with everyday life or to attract attention of close relatives (2). On the other hand, it has been postulated that the positive social standpoint towards the sick role helps reinforce the act (8).

The prevalence of factitious disorders is higher in lower socio-economic backgrounds. This has been traced to a lesser acceptance of psychological illness in these communities that prompts the patient to mask them as physical ones when assuming the sick role or asking for help (9). These disorders are more prevalent in females as well (10).

Childhood abuse and trauma are one of the predictors for emergence of a vast array of psychosomatic disorders (11). This patient had experienced physical abuse in her childhood too (according to her own words which could through no other means be verified). A study proposed that exposure to acute stressors, especially recent loss of close relatives, as we can see in this patient, can trigger the emergence of such disorders (12). 

The possibility of a patient with somatoform or factitious disorder having a seriously ill parent in childhood has been reported to be three times higher than having none (13). However, no history supporting the learning theory etiologies was found in this case. Attention-seeking is also cited as an etiology for these disorders (14), which was clearly cited as the main reason for the act by the patient.

Marital problems were also found that support the idea of some studies considering them as one of the triggers of factitious disorders (15). This notifies that family and couple therapy should be included in our therapeutic programs.

Factitious disorders usually are accompanied by anxiety and depression (2, 10). Anti-depressant drug administration has been shown to be helpful (2). Same is true about psychotherapies working mostly on these emotional disturbances. The presence of major depression and symptoms of anxiety in this case shows the presence of these disorders and shows why these measures help decrease the symptoms. 

Psychodynamic approaches and confrontation have shown rather incapable of improving the symptoms in a variety of psychosomatic disorders (10, 16). Supportive therapy on the other hand, has had significant results in the treatment of factitious disorders (2, 10, 14). As noted above, it was helpful in the management of the case.

## Conclusion

When encountering chronic vomiting, the possibilities are psychiatric causes or prolonged medication. If the latter is not the case, looking for the presence of depression and anxiety symptoms, signs of unusual behavior or recent or childhood traumatic experiences can help identify psychiatric causes such as Münchausen’s syndrome.

## Authors' contributions

MZ conceived and designed the evaluation and drafted the manuscript. RB participated in designing the evaluation. MH-S re-evaluated the clinical data and revised the manuscript. All authors read and approved the final manuscript.

## Notes

**Declaration of interest**: None.
